# Musculoskeletal Disorders in the Clinical Practice of Dental Hygienists and Dentists, Prevention and Awareness among Italian Professionals: Focus on Enlarging Systems

**DOI:** 10.3390/clinpract14050150

**Published:** 2024-09-12

**Authors:** Andrea Butera, Carolina Maiorani, Giulia Fantozzi, Francesca Bergamante, Matteo Castaldi, Roberta Grassi, Cinzia Leuter, Andrea Scribante, Gianna Maria Nardi

**Affiliations:** 1 Unit of Dental Hygiene, Section of Dentistry, Department of Clinical, Surgical, Diagnostic and Pediatric Sciences, University of Pavia, 27100 Pavia, Italy; andrea.scribante@unipv.it; 2 Dental Hygienist Freelancer, Via Mazzini 141, 06121 Perugia, Italy; fantozzigiulia50@gmail.com; 3 Department of Clinical and Experimental Medicine, University of Pisa, 56126 Pisa, Italy; bergamantefrancesca11@gmail.com; 4 RDH DHA, ATASIO (Accademia Tecnologie Avanzate nelle Scienze di Igiene Orale), 70121 Bari, Italy; castaldi.matteo@libero.it; 5 Department of Oral Surgery, Tor Vergata University, 00100 Rome, Italy; grassi.roberta93@gmail.com; 6 Medical Statistics and Molecular Epidemiology Unit, Campus Biomedico University of Rome, 00042 Rome, Italy; c.leuter@unicampus.it; 7 Unit of Orthodontics and Paediatric Dentistry, Section of Dentistry, Department of Clinical, Surgical, Diagnostic and Paediatric Sciences, University of Pavia, 27100 Pavia, Italy; 8 Department of Dental and Maxillofacial Sciences, “Sapienza” University of Rome, 00161 Rome, Italy; giannamaria.nardi@uniroma1.it

**Keywords:** musculoskeletal disorders, clinical practice, posture, operation, dental hygienist, dentist

## Abstract

(1) Background: Musculoskeletal disorders of the upper limbs are a common medical condition among dental hygienists and dentists, making them a common occupational risk for dental professionals. The purpose of the work was to collect information about the dental professions and their habits to highlight what can be good practices to be carried out to prevent any musculoskeletal disorders. (2) Methods: To identify habits and problems inherent in the professional activity of dental hygienists and dentists, a questionnaire was formulated on the use of enlarging systems. (3) Results: The questionnaire was completed by 241 dental professionals. As for the use of magnifiers, 72.6% of participants use them: among dental hygienists, 67.8% use magnifiers, among dentists, 80.9% use magnifiers; there is a statistically significant difference. There is no statistically significant difference between professions regarding muscle disorders. (4) Conclusions: For a clearer assessment, it would be appropriate to submit the questionnaire to a wider sample of professionals, to define better the correlation between musculoskeletal disorders, work activity and the type of enlarging systems used.

## 1. Introduction

Musculoskeletal disorders of the upper limbs are a common medical condition among dental hygienists and dentists [1], making them a common occupational risk for dental professionals [2]. The nature of dental work requires the repetitive movement of the arms, wrists and hands while taking prolonged and uncomfortable positions for the trunk, neck and shoulders [3].

Musculoskeletal disorders (MSD) due to repetitive movements and biomechanical overload of the upper limbs [4] are a cause of disability in the working population [5]. In this sense, it is necessary not only to carefully assess these risks in work activities with repetitive movements but also to correctly apply the criteria of ergonomics [6]. The correct positioning of the operator and the patient assumes a fundamental role in an efficient clinical treatment [7], to prevent any damage to the patient and limit the disturbances of the musculoskeletal system of the operator [8].

The main occupational diseases of ergonomic origin are closely related to the postural needs of the different professions [9], especially for those requiring specific measures for the prolonged maintenance of non-physiological positions [10], the use of dedicated instruments [11] or the stress of muscular or sensory functions [12].

Maintaining a correct working posture, therefore, allows good biomechanical functionality of the whole body during all activities, which will result in the professional achieving a psychophysical balance to the total benefit of them and the patient undergoing treatment [13]. On the contrary, not respecting the principles of an optimal posture may lead the professional to feel discomfort due to the professional effort that may result not only in a reduced and/or compromised work activity but could also be reflected in everyday life [14]. As with most professional pathologies of a postural nature, the most involved body segment is the spine, the main and functional axis of our entire organism [15]. For this reason, the ideal position during the exercise is aimed at preserving the correct curvatures of the spine, both at the cervical and dorsal levels and at the level of the lumbar lordosis, where the prolonged maintenance of incorrect postures can easily result in highly disabling pathologies such as lumbago and chronic lumbosciatalgia [16].

Moreover, for dental hygienists/dentists, a complete and accurate vision of the operating field is necessary to maintain a front inclination of the head that sufficiently remains between 15 and 20 degrees [17], avoiding neck muscle tension and visual fatigue [18].

The use of the enlarging systems limits distortions and blurring according to the binocular vision of the human eye and allows it to work, even for prolonged periods, reducing visual fatigue, also thanks to the possibility of preparing graduated and personalized lenses [19]; acts as a barrier of defence, protecting the eyes from blood and liquids contaminants or abrasives [20]; forces the operator to maintain a predefined working posture, correct, effective and not stressful, which provides relief to the spine: neck and head maintain a more balanced position, reducing the fatigue of the stabilizer muscles of the shoulders [21].

This arrangement facilitates the alignment of the shoulders with the hips and floor, as well as ensuring that the elbows are in accordance with the remainder of the body while maintaining a straight line for the forearms and wrists. It is essential that the body’s weight is evenly distributed on the stool, allowing for a gap of approximately 3 cm between the edge of the seat and the popliteal fossa of the knees, which should remain slightly apart, with the feet firmly positioned against the floor [22]. The operator’s ability to maintain an appropriate posture is inherently influenced by their relative positioning in relation to the patient; specifically, the patient’s oral cavity should be aligned with the professional’s elbow, although this position may be affected by actual access to said oral cavity [23]. By conceptualizing the practitioner’s position around the patient as if mapped into a clock face with the patient at its center, it can be observed that a right-handed practitioner typically orients themselves within the hours of 8:00 to 13:00 during clinical procedures, whereas a left-handed practitioner will occupy positions between 11:00 and 16:00 [24]. The operating field is centered around the oral cavity of the patient, where the operative field means the dental chair with the patient, the unit containing the instrumentation, which allows rapid accessibility to the working tools. The essential element for access and visibility during treatment is given by the flexibility of movements of the professional stool, by adequate lighting reinforced by the use of an enlargement. It is, therefore, clear how the correct instrumentation used by the professional can largely influence the posture he assumes during the treatment of the patient [25].

The practice of dental hygiene or dentistry necessitates daily engagement with various ergonomic and postural considerations associated with the positions adopted during operational phases, the instruments employed, and the sequences of movements required [26]. If these aspects are not addressed with appropriate precautions, they may lead to the development of various musculoskeletal disorders [27]. Both the upper and lower limbs, as well as the spine, can be affected in distinct ways by conditions that are preventable and treatable through targeted interventions [10]. Disorders impacting the upper limbs are primarily associated with the instruments used, which do not always adequately accommodate ergonomic requirements, alongside repetitive or uncomfortable movements; these include carpal tunnel syndrome, flexor tendonitis of the fingers, epicondylitis, rotator cuff tendonitis, cervical pain, lumbar pain, disc protrusions, and hernias. Furthermore, improper positioning of the lower limbs can result in debilitating occupational diseases often linked to the overall condition of the lumbar spine [28]. Consequently, it is imperative for practitioners to adopt a proper seated posture that provides both stability and freedom of movement around the patient while employing ergonomic precautions that align with their professional operational needs [12].

In Italy, according to a recent study, 59.9% of professionals suffer from work-related neck pain, 52% in the lumbar area, 43.3% in the shoulders, 37.7% in the dorsal area but that also wrists, elbows and ankles are affected by algic problems. The incidence of the problems is proportional to the number of hours of outpatient activity, with a higher incidence for 30–40 h per week, to the number of years of professional activity, with a higher incidence for operators in activity for 21–40 years [27].

The objective of this study was to gather information regarding the dental professions and their practices in order to identify effective strategies that may be implemented to prevent musculoskeletal disorders, such as the use of enlarging systems, that ensure a correct position of the operator towards the patient and consequently reduce the visual and muscular stress [19]. The research question aims to assess whether the use of enlarging systems improves work-related visual and muscular disorders.

## 2. Material and Methods

A cross-sectional study was conducted in the form of an online questionnaire using the platform Google Forms (Unit Internal Review Board approval: 2022-0518 University of Pavia).

In order to identify habits and problems inherent in the professional activity of dental hygienists and dentists, a questionnaire was formulated on the use of enlarging systems (Table 1). The questionnaire was distributed among dental professionals and designed to examine their working habits in order to evaluate the potential correlation between the use of enlarging systems and the occurrence of muscular and/or visual disorders.

The questions focused on knowledge of gender, age, profession, years of work, muscular and/or visual problems existing or occurring over the years and the use of the enlarging systems during working hours [27].

The questionnaire was distributed to students and professionals in the fields of dental hygiene and dentistry, with the exclusion of retired practitioners and those individuals who did not complete the questionnaire accurately (less than 90% of the questions were answered). The questionnaire covers gender, age and profession (both years of work and specialization), musculoskeletal and/or visual disorders, stretching, physical activity (and frequency), working and sitting positions, use and type of enlargers (including the stages in which they are used).

Sample size (Alpha = 0.05, power = 95%) was calculated. The assumptions were based on previous findings [29], with an expected frequency of musculoskeletal disorders of 10.5% in the normal population and an expected growth of 8% in the dental professional population [30]. Therefore, 241 questionnaires were needed for the study.

### Statistical Analysis

Descriptive statistics were used to describe sample characteristics. Frequency and percentage mean and standard deviation were used to describe categorical variables and continuous variables, respectively. Absolute frequencies were used and calculated (number of times a variable mode occurs; absolute frequency, considered X as variable, collected on a sample of x statistical units and which can be manifested in k possible ways v_1_, v_2_, …, v_k_, is v_i_, with 1 ≤ i ≥ k natural number between 0 and the total number of statistical units, so 0 ≤ f (v_i_) ≥ n); relative frequencies were used and calculated [f_r_ (v_i_) = $\frac{f\;\left(vi\right)}{n}$, decimal number between 0 and 1, so 0 ≤ f (v_i_) ≥ 1)]; percentage frequencies [f_r_ (v_i_) = (f_r_ (v_i_)·100)%]. The arithmetic average (μ = $\frac{1}{N}\overset{N}{\underset{i=1}{\sum }}xi$) and standard deviation [σ = $\frac{\sqrt{\sum_{i=1}^{N}\left(xi-\mu \right)}y}{N}\;$] were also calculated based on the variables considered for statistical analysis.

The χ^2^ test was used to verify the associations between categorical variables. Statistical tests were two-tailed and statistical significance was set to *p*-value < 0.05. Data were coded on an electronic sheet and statistical analysis was performed with SPSS v.19 (IBM Corp., Armonk, NY, USA).

## 3. Results

The questionnaire was completed by 241 dental professionals, 147 females and 94 males; 14.5% of them were under 30 years of age, 46.5% were between 31 and 46 years old and 38.6% were over 46 years old (Table 2). Age, occupation and gender did not show any significant influence on the results (*p* > 0.05).

Table 3 and Table 4 show the types of magnification systems used and the times of use in professional practice. Dentists have been using loupes for many years and the difference is statistically significant (*p* = 0.000); it should be remembered that dentists are on average older and have a greater number of years of professional experience. Among the dental hygienists, 67.8% use magnifying glasses, and among dentists, 80.9% use magnifying glasses. There is a statistically significant difference (*p* = 0.000).

In total, 71.4% of the study’s population suffer from visual disorders and 31.5% from muscle disorders. Dentists are more present among those with visual disorders (79.8%) than hygienists (66.4%). There is a statistically significant difference (*p* = 0.000). Dental hygienists have more muscular problems (32.9%) than dentists (29.2%). The difference is not statistically significant (*p* = 0.553). The percentage of muscular and visual problems would seem to be related to the years in the profession. In general, in the population studied, the use of magnification systems was found to cause more visual problems than muscular problems, where the percentage of people who suffer from them is significantly lower than that of people who do not use them, although the difference is not statistically significant (Table 5).

The population was 152 dental hygienists and 89 dentists; the average age of the dentists was older, and this is a possible confounding factor for other analyses. In total, 35.3% of the population object of the study had carried out the profession for under 10 years, 33.6% for 11 to 20 years and 30.3% for over 20 years (Table 2). Dentists were most present in the n-class of higher professional years with a significant difference (*p* = 0.000). Dentists used more magnifying systems, compared to dental hygienists and for more years (since it can be linked to the fact that they work longer and have a higher age). Both use them for most of their work (Table 6).

## 4. Discussion

It is clear that the main occupational diseases of ergonomic origin are closely related to the postural needs of the different professions, especially those that require specific measures for the prolonged maintenance of non-physiological positions [2], the use of dedicated instruments or the stress of muscular or sensory functions (for example sight) [12]. With regard to the practice of dental hygiene and dentistry, it is typically observed that the professional operates from a neutral position with optimal access, where adequate lighting and visibility significantly enhance procedural effectiveness. The patient is positioned in a manner that facilitates the professional’s work, thereby allowing for an efficient and brief appointment while fostering a strong rapport between both parties involved [22]. It is essential to acknowledge that the advantageous effects of an ideal working environment for the practitioner extend beyond their own health; by streamlining their tasks, such conditions ultimately yield improved outcomes and tangible benefits for the patient. [24].

The population covered by this study is fairly heterogeneous by gender, age and profession: 241 professionals (63.1% dental hygienists, 36.9% dentists) answered the questionnaire, mainly over 30 years of age (61% females, 39% males). The questionnaire submitted shows that the older population is that of dentists, who have been working for several years and who suffer more from visual impairments, compared with dental hygienists (where muscular disorders prevail; there is no statistically significant difference). In addition, dentists were using more enlarging systems in the practice and for much longer; this is due to the fact that they are older and have more professional years than dental hygienists.

From the results obtained by administering the proposed questionnaire, it can be inferred that the use of enlarging systems (used mostly by dentists) results in a low percentage of work-related muscle disorders (31.5%). These data would require further analysis; it would be appropriate, in fact, to verify effectively the working position adopted by professionals, although they use enlarging systems. The different ages, genders and work experience of the population surveyed may have influenced the reported results. For example, with gender, women (the population predominant in this study) develop more musculoskeletal disorders, probably due to reduced muscle mass [31], hormonal changes/stress [32] and increased incidence of osteoporosis (age-related problems) [33]. Based on the age and work experience (number of years in the job) of the population studied, operators who work long hours per day favor the onset of musculoskeletal problems [34]; it can, therefore, be concluded that those who have worked for many years are more susceptible to these problems [35].

Several studies have shown that the use of enlarging systems allows assuming a correct posture (as required), improving work ergonomics and reducing musculoskeletal disorders [36] in addition to optimizing diagnostic accuracy and procedural quality, with benefits for both professionals themselves and patients [37]. These statements are also confirmed by the results of our questionnaire, where those who use enlarging systems have fewer muscle problems (69.6% of respondents do not suffer from muscle problems).

In addition, the use of magnifiers allows the activity to be carried out at a greater working distance [19], resulting in less visual fatigue [20]; this would not agree with the results of this study, which sees a greater percentage of visual disturbances among dentists, these are the largest users of the enlarging systems (according to the population studied). The results of this study suggest that postural imbalances in the practice of the dental hygienist may contribute to the numerous pathologies found in the operator: the use of targeted orthopedic aids and enlarging systems contribute significantly to the correction or, even better, the prevention of many disorders [27]. The use of ergonomic chairs or saddles and magnification systems would seem to favor working positions that reduce or alleviate pain in the shoulders, arms and hands [38]. With regard to neck pain, a recent literature review states that the effect of magnification systems on reducing pain in the neck region is poor [36]; however, it can be concluded that the use of a lighting system incorporated into magnification systems may lead to a better field of vision [39], reducing neck flexion [40].

In addition, the instrumentation used also affects musculoskeletal disorders: the use of instruments with a diameter of 10 mm and weight of 10 g or less is recommended; a handle with a diameter between 6 and 8 mm requires a substantial muscular force to control the function, prolonged use may, therefore, lead to an overloading of the tendons and muscles, both extrinsic and intrinsic, of the hand [41].

Strategies to be adopted in the protection of the health of operators must include education on the correct planning of work and prevention of musculoskeletal disorders. Professional ergonomics can guide the operators’ choices on a suitable tool and its practical use, frequently alternating tools of different diameters depending on the type of result, without neglecting the search for the best working position [42]; the position is also affected by the use of magnifying systems to relieve neck pain and improve posture [1].

Therefore, the training should address the identification of work practices related to musculoskeletal disorders, their risk factors and principles of self-assessment.

The principal limitation of this study appears to be the heterogeneity of the sample previously described, particularly concerning factors such as gender, age, years of experience, and the utilization of various types of magnification systems. This variability hinders a comprehensive elucidation of how magnification systems contribute to the alleviation of musculoskeletal disorders. It would also be prudent to standardize the sample in order to identify additional strategies that may effectively mitigate this issue.

Furthermore, the questionnaire was designed to ascertain the habits of dental hygienists and dentists, with the aim of understanding their influence on the development of musculoskeletal conditions. However, this data collection does not yield conclusive evidence regarding risk or causal factors associated with these disorders based on the results obtained. To address this gap, an analysis of professionals’ physical health prior to their employment should be conducted, allowing for comparisons of any changes over their years in practice. Such data ought to be correlated and interpreted in relation to variables such as age, daily working hours, and usage percentages of magnifying systems. Training should emphasize sound ergonomic practices and promote a healthy lifestyle that includes regular physical activity.

Despite the limitations, the use of this questionnaire provides an overview of the habits of dental hygienists and dentists (in Italy) and how these, together with the work, could affect muscular and visual disorders. Moreover, it is imperative that we institute and assess proactive measures to guarantee sufficient postural training throughout the university years and the initial stages of clinical practice, with the objective of mitigating disorders over time through preventive strategies.

Further research is needed to determine new postural techniques and higher quality aids for dentists and dental hygienists, in order to reduce musculoskeletal disorders.

It would be useful to carry out studies involving dental hygienists and dentists, trying to identify the aids that improve and reduce MSDs, such as working positions influenced by chairs, lighting and magnification systems, and trying to standardize the sample by age, years of work and use of such aids, in order to obtain more accurate results.

## 5. Conclusions

Musculoskeletal disorders, due to repetitive movements and biomechanical overload of the upper limbs, are a cause of disability in the working population of the dental sector. In this regard, it is essential to meticulously evaluate the risks associated with work activities that involve repetitive movements, as well as to appropriately implement ergonomic principles.

In addition, the use of enlarging systems helps to assume a correct working position and could help to reduce the disorders, especially muscle disorders, related to the profession.

However, the data collected from the questionnaire administered to dental professionals (and students) are not sufficient to clarify whether the use of enlarging systems can reduce visual and muscular disorders: the population examined is heterogeneous in terms of gender, age, years of employment and use of them. Therefore, for a clearer assessment, it would be appropriate to submit the questionnaire to a wider sample of professionals to better define the correlation between musculoskeletal disorders, work activity and the type of enlarging systems used.

## Figures and Tables

**Table 1:** Questionnaire data was collected as *x*_1_, *x*_2_, …, *x_N_*, which are the values of the character *X* observed on the *N* units of the collective.

Question	Answer
Gender	☐ Male ☐ Female
Age	
You are currently	☐ Student ☐ Professional ☐ Altro:
What profession are you in?	☐ Dental Hygienist ☐ Dentist
If you are a dentist, which branch do you focus on (as many answers as possible)?	☐ Endodontic dentistry ☐ Prosthetic dentistry ☐ Surgery ☐ Conservative dentistry ☐ Orthodontics ☐ Gnathology
How many years have you been working?	
Do you have visual disorders?	☐ No, none ☐ Yes, presbyopia ☐ Yes, short-sightedness ☐ Yes, hyperopia ☐ Yes, astigmatism ☐ Yes, squint (cross-eyed) ☐ Yes, amblyopia (lazy eye) ☐ Yes, maculopathy ☐ Other:
Do you have any musculoskeletal disorders?	☐ Yes ☐ No
If so, which one?	
Do you do stretching exercises or practice muscle relaxation?	☐ Yes ☐ No
If so, when do you usually perform them:	☐ Between one patient and another ☐ At the end of the day ☐ When I have time ☐ Other
During work:	☐ Always stay in the same place as the patient’s head ☐ Rotate around the patient’s head to better perform tasks (clock) ☐ Other:
Do you change positions during the work session?	☐ No, I always sit ☐ No, I always stand ☐ Yes, alternate sitting/standing
What chair do you use?	☐ Traditional seat ☐ Saddle seat ☐ Shoulder ☐ Other:
Do you play any sports?	☐ Yes ☐ No
If so, which one?	
If so, how often?	
Do you use magnifiers?	☐ Yes Reasons for the reply ☐ No Reasons for the reply
If only in some cases, specify in which work steps	
When and how did you learn about the enlarging systems?	☐ During the course of the degree ☐ After the course of the degree ☐ Congresses/courses ☐ Social groups dedicated to the profession ☐ Other:
If so, how long have you been using them?	
If so, what kind of magnifiers do you use?	☐ Galileian ☐ Personalized Galileian (TTL) ☐ Prismatic ☐ Custom Prismatic (TTL) ☐ Expanding bands ☐ Other:
You use:	☐ Magnifying glasses Reasons for the reply ☐ Helmet Reasons for the reply ☐ Altro:
What magnification do you use (galilean system)?	☐ 2× ☐ 2.5× ☐ 2.8× ☐ 3.5× ☐ 4×
What magnification do you use (prismatic system)?	☐ 3.5× ☐ 4× ☐ 5× ☐ 6×
Did you evaluate the parameters before purchasing the enlargement system?	☐ Yes ☐ No
If so, what (more answers possible)?	☐ Magnifying ☐ Working distance ☐ Depth of field of view ☐ Interpupillary distance ☐ Work area ☐ Angle of optical declination ☐ Angle of convergence ☐ Lighting
How tall are you?	
Do you know your working distance? (distance between operator’s eye and work area)	☐ Yes ☐ No
If so, what is it?	
You use the magnifiers:	☐ During the whole working life ☐ Only in a few stages Specify which:
Did you visit before using the magnifiers?	☐ Yes, an orthotic visit ☐ Yes, an ocular sight ☐ Yes, an optometric visitor ☐ No
Before you started using magnifiers, did you already suffer from visual disorders? (more answers possible)	☐ No, none ☐ Yes, presbyopia ☐ Yes, short-sightedness ☐ Yes, hyperopia ☐ Yes, astigmatism ☐ Yes, squint (cross-eyed) ☐ Yes, maculopathy ☐ Other:
Do you think the use of magnifiers has reduced visual fatigue?	☐ Yes ☐ No ☐ Other:
Since using the magnifiers, you have noticed:	☐ Improvement of posture ☐ Improvement of musculoskeletal disorders ☐ Improvement of both (posture and musculoskeletal disorders) ☐ Worsening of the posture ☐ Worsening of musculoskeletal disorders ☐ Worsening of both (posture and musculoskeletal disorders) ☐ Nothing different ☐ Other:
You disinfect the magnifiers?	☐ Yes, with every patient change ☐ Yes, at the end of the day ☐ No, never ☐ Other:
If yes, you disinfect:	☐ Only the lenses ☐ All components of the enlarger ☐ Other:
Do you put the visor on top of the magnifiers?	☐ Yes ☐ No Reasons for the reply
Do you use the front illuminator?	☐ Yes ☐ No

**Table 2:** Respondent’s characteristics (n = 241).

Gender	Frequency	Percentage
Females	147	61.0
Males	94	39.0
Age (years)		
<30	35	14.5
31–46	112	46.5
>46	93	38.6
Missing	1	0.4
Profession		
Dental Hygienist	152	63.1
Dentist	89	36.9
Years of occupation		
<10	85	35.3
11–20	81	33.6
>20	73	30.3
Missing	2	0.8

**Table 3:** Use of magnifiers according to type of system.

Type of System (n = 190) *	Frequency	Percentage
Galileian	59	24.5
Personalized Galileian(TTL)	58	24.5
Prismatic	47	19.5
Custom Prismatic (TTL)	15	6.2
Expanding bands	11	4.6

* total for users may vary due to missing data.

**Table 4:** Use of magnifiers according to type of system based on the “×” scale.

Type of System (n = 204) *	Frequency	Percentage
Galileian system (n = 131)		
2×	67	51.1
2.5×	20	15.3
2.8×	3	2.3
3.5×	28	21.4
4×	13	9.9
Prismatic system (n = 73)		
3.5×	11	15.1
4×	42	57.5
5×	12	16.4
6×	8	11.0

* total for users are higher because more than one answer was allowed.

**Table 5:** Profession, years of occupation and use of magnifiers according to medical conditions.

	Medical Conditions					
	Visual Disorders			Muscular Disorders		
Total N = 241 *						
	**Yes**	**No**	***p*** -Value **	**Yes**	**No**	***p*** -Value **
Profession, n (%)						
Dental Hygienist	101 (66.4)	51 (33.6)	0.027	50 (32.9)	102 (67.1)	0.553
Dentist	71 (79.8)	18 (20.2)		26 (29.2)	63 (70.8)	
Years in occupations, n (%)						
<10	53 (62.4)	32 (37.6)	0.003	18 (21.2)	67 (78.8)	0.028
11–20	56 (69.1)	25 (30.9)		29 (35.8)	52 (64.2)	
>20	63 (86.3)	10 (13.7)		29 (39.7)	44 (60.3)	
Use of magnifiers, n (%) ***						
Yes	140 (73.3)	51 (26.7)	0.195	58 (30.4)	133 (69.6)	0.445
No	32 (64.0)	18 (36.0)		18 (36.0)	32 (64.0)	

* total for each variable may vary due to missing data, ** χ^2^, *** “yes” and “in some cases” were united.

**Table 6:** Age, years of occupation according to profession, use of magnifiers during work and duration of use according to profession.

	Profession		*p*-Value **
Total (N) = 241 *	Dental Hygienist	Dentist	
Age in years, n (%)			0.000
<30	31 (20.5)	4 (4.5)	
31–46	73 (48.3)	39 (43.8)	
>46	47 (31.1)	46 (51.7)	
Years in occupation, n (%)			
<10	67 (78.8)	18 (21.2)	0.000
11–20	54 (66.7)	27 (33.3)	
>20	29 (39.7)	44 (60.3)	
Use of magnifiers, n (%)			0.000
Yes	103 (67.8)	72 (80.9)	
In some case	7 (4.6)	10 (11.2)	
No	42 (27.6)	7 (7.9)	
Use of magnifiers during work, n (%)			0.000
During the whole working life	93 (86.1)	60 (75.0)	
Only in a few stages	15 (13.9)	20 (25.0)	
Duration of use magnifiers, n (%)			0.000
<5	64 (58.2)	13 (15.9)	
6–15	39 (35.5)	36 (43.9)	
16–25	5 (4.5)	21 (25.6)	
>25	2 (1.8)	12 (14.6)	

* total for each variable may vary due to missing data, ** χ^2^.

## Data Availability

The original contributions presented in the study are included in the article, further inquiries can be directed to the corresponding author.
